# Negative BOLD response to interictal discharges in epilepsy using intracranial EEG-fMRI

**DOI:** 10.1016/j.ynirp.2026.100393

**Published:** 2026-08-06

**Authors:** Jessie Hart Szostakiwskyj, Victoria Mosher, Perry Dykens, William Wilson, Yi Fan Qi, Dan J. Pittman, Laura Gill, Joseph Peedicail, Brad G. Goodyear, Paolo Federico, on behalf of the, Calgary Comprehensive Epilepsy Program collaborators, Yahya Aghakhani, Walter Hader, Nathalie Jetté, Colin Josephson, Karl Martin Klein, William Murphy, Neelan Pillay, Andrea Salmon, Shaily Singh, Yves Starreveld, Samuel Wiebe

**Affiliations:** a Hotchkiss Brain Institute, Cumming School of Medicine, University of Calgary, Canada; b Seaman Family MR Research Centre, Cumming School of Medicine, University of Calgary, Canada; c Department of Clinical Neurosciences, Cumming School of Medicine, University of Calgary, Canada; d Department of Radiology, Cumming School of Medicine, University of Calgary, Canada

**Keywords:** Epilepsy, EEG, Intracranial EEG, Functional MRI, Negative BOLD

## Abstract

**Introduction:**

Combined electroencephalography and functional magnetic resonance imaging (EEG-fMRI) identifies regions of neural activity in response to interictal epileptiform discharges (IEDs). While positive BOLD responses have historically been used to localize epileptogenic tissue, negative BOLD responses remain poorly understood. This study examined the spatial relationship between positive and negative BOLD responses to IEDs and their associations with epileptogenic tissue and the default mode network (DMN) using intracranial EEG-fMRI.

**Methods:**

70 adult participants (57 met inclusion criteria), with drug-resistant focal epilepsy, underwent intracranial EEG-fMRI during presurgical monitoring. BOLD response maps were generated and maximum positive and negative clusters identified. Linear mixed models examined distances between BOLD clusters and both IED-generating contacts and contacts closest to clinically-defined seizure onset zone. A subset of 38 participants were assessed for spatial overlap and functional connectivity with the DMN.

**Results:**

Maximum positive BOLD clusters were significantly closer to both IED-generating contacts (estimate = −30.0, 95% CI [-55.5, −4.37]) and seizure onset zone contacts (estimate = −32.7, 95% CI [-57.5, −8.43]) compared to maximum negative clusters, when the absolute maximum is positive. Negative BOLD clusters demonstrated significantly greater spatial overlap with the DMN than positive clusters (22.5% vs 10.0%, p = 0.04).

**Conclusions:**

Positive BOLD responses provide superior localization of epileptogenic tissue compared to negative responses in intracranial EEG-fMRI. In contrast, negative BOLD clusters show greater spatial overlap with the DMN, suggesting they may reflect network deactivation at the time of IED events. These findings support prioritizing positive BOLD responses for clinical decision-making in presurgical epilepsy evaluation.

## 1 Introduction

The primary goal of epilepsy treatment is seizure freedom, yet approximately 30% of individuals with epilepsy cannot achieve this goal using anti-seizure medications alone (Sultana et al., 2021). Patients with drug-resistant focal epilepsy may be candidates for resective surgery to remove the brain region where seizures arise, known as the seizure onset zone (SOZ) (Noachtar and Borggraefe, 2009). Prior to surgery, a variety of imaging and diagnostic tests are used to better delineate the SOZ. Combined electroencephalography and functional magnetic resonance imaging (EEG-fMRI) has been proposed as one such method to help identify the SOZ in patients with drug-resistant focal epilepsy.

EEG-fMRI identifies regions of increased neural activity by measuring relative changes in the blood oxygen level-dependent (BOLD) signal of fMRI in response to interictal epileptiform discharges (IEDs) detected on EEG (Khoo et al., 2017; Arafat et al., 2025; Tehrani et al., 2021). Historically, the positive BOLD response to IEDs, representing a relative increase in the BOLD signal, has been used to localize the SOZ in epilepsy patients (Wilson et al., 2024; Zijlmans et al., 2007; Chauvière et al., 2012). The negative BOLD response, characterized by a relative decrease in the BOLD signal, was traditionally considered vascular in nature and not reflective of meaningful brain function (Arteaga et al., 2014; Harel et al., 2002). However, recent studies have begun exploring neuronal-based theories of the negative BOLD response, suggesting that negative BOLD signals may have different underlying mechanisms, reflecting inhibition or reduction of brain activity rather than vascular phenomena (Sten et al., 2017; Mullinger et al., 2014; Jacobs et al., 2014; Pasley et al., 2007; Goense et al., 2012; Shmuel et al., 2002, 2006; Fracasso et al., 2022; Tal et al., 2026; Gouws et al., 2014; Boorman et al., 2010; Allison et al., 2000; Kastrup et al., 2008; Klingner et al., 2010; Jorge et al., 2018; Schafer et al., 2012). Furthermore, neurovascular coupling may differ between BOLD signal increases and decreases between cortical layers (Goense et al., 2012), and they may have specific laminar profiles (Boorman et al., 2010; Fracasso et al., 2018; Huber et al., 2014, 2015) with timing that can vary depending on the task (Huber et al., 2014). In contrast, optogenetic stimulation of cortical inhibitory GABAergic neurons can increase local cerebral blood flow while decreasing local neuronal activity (Anenberg et al., 2015).

Negative BOLD responses during task performance are commonly found in regions that overlap with the default mode network (DMN), also known as the task-negative network. The DMN encompasses brain regions that are active during rest and deactivated during task-focused states (Poerio et al., 2017; Szinte and Knapen, 2020; Marino et al., 2019). In this context, the negative BOLD response while performing a task may represent, or be related to, DMN deactivation while task-related networks dominate. Disruptions in DMN function have been observed within the mesial temporal lobe and hippocampus in patients with epilepsy (Liao et al., 2011; Kobayashi et al., 2006a), and DMN dysfunction has also been correlated with seizure duration in idiopathic generalized epilepsy (McGill et al., 2012; Archer et al., 2003). Furthermore, reduced DMN connectivity has been linked to both epilepsy severity and medication resistance (Kay et al., 2013), suggesting a fundamental role of network-level dysfunction in epilepsy pathophysiology, and the possibility for negative BOLD to capture this dysfunction.

Despite these observations, the relationship between negative BOLD responses to IEDs, the DMN, and epilepsy pathophysiology remains poorly defined. For example, during seizures in rats, positive BOLD signal increases were seen in the cortex, but BOLD signal decreases were seen in the hippocampus, suggesting that the nature of ictal BOLD signal changes may be site-dependant (Schridde et al., 2008). Human studies using scalp EEG-fMRI demonstrate that the maximum negative BOLD responses to IEDs often overlap with DMN nodes. This overlap has led to some groups excluding negative BOLD clusters that overlap with the DMN from their localization studies (Khoo et al., 2017; Koupparis et al., 2021). It has also been proposed that the absolute maximum response to IEDs, regardless of polarity, provides the best localization of the SOZ (Jacobs et al., 2014; Archer et al., 2003), suggesting that negative BOLD responses may also indicate epileptic brain activity rather than solely reflecting DMN deactivation. However, to our knowledge the localization value of negative BOLD has not been directly tested as compared to positive BOLD. Additionally, these studies utilized scalp EEG-fMRI, which is limited in its ability to spatially localize IED generation. The use of intracranial EEG-fMRI (iEEG-fMRI) offers several advantages over scalp recordings, including more precise IED detection and substantially higher IED occurrence rates. However, intracranial electrodes are spatially limited to the presumed SOZ region, and the implanted electrodes create MRI artifacts that may obscure nearby BOLD signals (Wendling et al., 2003; Tao et al., 2007; Fernández and Loddenkemper, 2013). These methodological differences between scalp and intracranial approaches necessitate separate investigation of negative BOLD responses in the intracranial setting.

The aim of the present study is to differentially examine positive and negative BOLD responses to IEDs using intracranial EEG-fMRI in patients with drug-resistant focal epilepsy. Given that negative BOLD seems to reflect deactivation or potentially DMN involvement, we hypothesized that maximum positive BOLD responses would be more spatially concordant with both the IED-generating EEG contacts and the SOZ compared to maximum negative responses. We further hypothesized that negative BOLD responses would demonstrate greater spatial overlap and functional connectivity with the DMN than positive BOLD responses.

## 2 Methods

### 2.1 Participants

Seventy adult participants with drug resistant focal epilepsy admitted for pre-surgical inpatient intracranial video-EEG monitoring were recruited. Participants were recruited using the following criteria: focal epilepsy, at least 18 years of age, no MRI contraindications, and no severe post-electrode implantation complications (e.g., subdural hematoma, infection, intracranial hemorrhage). This study was approved by the University of Calgary Conjoint Health Research Ethics board (REB20-0384). Written informed consent was obtained from all participants prior to participation.

Relevant demographic and clinical information were collected from patient charts including the duration of epilepsy, frequency of seizures, previous surgical resection, and location(s) of IEDs and the SOZ. The SOZ contacts were defined as those contacts involved in ictal onset during intracranial video-EEG monitoring. The geometric centre of all SOZ contacts was used as the location of the SOZ, calculated along each of 3 axes with a general equation (Equation 1).

Equation 1: Geographic centre

$$X_{GC}=\frac{\left(X_{1}+X_{2}+\ldots X_{n}\right)}{n}$$

### 2.2 Data acquisition

Each participant underwent simultaneous iEEG-fMRI which included two structural MRI sequences and up to three 20-min fMRI runs, during which iEEG data were simultaneously recorded from the participant's implanted electrodes. Electrodes were connected to either a custom 2-tailed (participants 1-7) or 8-tailed intracranial electrode connector allowing up to 20 or 64 channels, respectively, of data collection (Neuroscan, Compumedics, Charlotte NC, USA). This technical constraint meant we needed to be selective of which electrodes to study. EEG data from inpatient intracranial video-EEG monitoring were reviewed by two experienced epileptologists (two of PF, LG, JSP) to select up to eight electrodes for use during each participant's simultaneous iEEG-fMRI study. EEG data were recorded at 10 or 20 kHz using a SynAmpsRT amplification/digitalization system and either Scan 4.4 (prior to 2020) or Curry 8 software (after 2020; Neuroscan, Compumedics, Charlotte NC, USA). For participants 25 and above, the EEG system clock was synchronized to the MRI scanner clock to ensure alignment of the gradient switching artifact with the EEG data. In our early studies (Subjects 1-24), the EEG system clock was not synchronized to the MRI scanner clock, and gradient switching artifacts were removed using standard EEG-fMRI analysis techniques involving subtraction of a moving average of TR-duration waveforms (Allen et al., 2000).

MRI data were collected using either a 3 T GE Sigma LX whole body scanner with a receive-only eight-channel phased-array head-receive/body transmit coil (participants 1-7) or a 3 T GE Discovery MR750 whole body scanner equipped with a 12-channel receive-only phased array head coil (GE Healthcare, Waukesha, WI). The MRI protocol included multi-slice anatomical imaging (spoiled gradient-recalled two-dimensional multi-slice sequence: echo time [TE] = 2.1 ms, repetition time [TR] = 150 ms, flip angle = 18°, 128 x 128 matrix, 24 slices, 0.94 x 0.94 × 5 mm), anatomical three-dimensional T1weighted imaging (TE = 3.8 ms, TR = 9.3 ms, flip angle = 12°, 24 cm field of view, 320 x 256 x 64 matrix, 0.47 x 0.47 × 2 mm), and fMRI (gradient-recalled echo planar imaging: TE = 30 ms, TR = 1500 ms, flip angle = 65°, 24 cm field of view, 64 x 64 matrix, with 24, 25, or 29 slices, 3.75 x 3.75 × 5 mm).

### 2.3 iEEG processing

iEEG data collected during the simultaneous iEEG-fMRI session were processed using inhouse software. Pre-processing included channel rejection, within electrode average re-referencing, a 2nd order Butterworth highpass filter at 0.1 Hz and a 2000th order FIR lowpass filter at 500 Hz, channel-by-channel slice-wise average artifact subtraction, and a modified PCA. Following artifact removal, channel data were decimated to a sampling rate of 2500 Hz.

#### 2.3.1 IED marking and contact selection

Processed iEEG data were reviewed by two qualified epileptologists (two of YA, PF, LG, CJ, KMK, JSP, AS, SS) who identified the timing and morphology of the IEDs as well as the contacts where they occurred. Discrepancies between reviewers were resolved in person by both reviewers. IEDs were grouped according to location and morphology and each group was labelled as a separate IED type.

For each IED type, the contact(s) with maximum amplitude in the referential montage and/or phase reversal in the bipolar montage were defined as the most active electrode contact(s). A maximum of three most active contacts were identified using these criteria, in some cases on separate electrodes. The anatomical coordinates of these contact(s) were determined visually using the participant's 3D anatomical images viewed in FSLView (3.2.0), and the geometric centre of those coordinates was calculated as an estimation of the electrographic IED origin.

### 2.4 fMRI data processing

All fMRI data processing was completed using the FMRIB Software Library (Functional Magnetic Resonance Imaging of the Brain Software Library, Oxford, England) (Woolrich et al., 2009; Smith et al., 2004; Jenkinson et al., 2012). Preprocessing steps included: brain extraction, motion correction, slice-timing correction, anatomical registration, high-pass temporal filtering (sigma = 50 s), and spatial smoothing with a 6.0 mm Gaussian kernel. Datasets with motion exceeding 1.5 mm were split into 2 segments after removing the frames containing the excessive motion. This process was performed recursively, and any resulting segment less than 5-min was removed from the analysis. FSL's multivariate exploratory linear optimized decomposition into independent components (MELODIC) was used to decompose each dataset into 60 components. These components were visually inspected by 2 reviewers (VM, WW) to identify components that could be attributed primarily to either noise or artifact that were subsequently regressed from the data. Difference between reviewers were resolved in-person by both reviewers and components attributed to noise were subsequently regressed from the data (Beckmann and Smith, 2004; Griffanti et al., 2017).

For each IED type in each patient, four hemodynamic response functions (time to peak offsets of 3, 5, 7, and 9 s) were convolved with the IED timings to create four regressors to predict the fMRI time course for each IED-type (see Fig. 2 for example time series). Statistical analyses of these models with the recorded fMRI data were performed using FEAT (FSL Expert Analysis Tool) and FILM (FMRIB Improved Linear Model) with local autocorrelation correction for each run (Woolrich et al., 2001). A statistical average of each fMRI run was generated using FLAME (FMRIB Local Analysis of Mixed Effects), employing a fixed effects model, forcing the random effects variance to zero (Beckmann et al., 2003) Z-score maps of statistically significant BOLD signal changes (BOLD response maps) using cluster correction with a cluster z threshold of z > ±3.1 and a cluster *p* threshold of 0.01 were generated for each convolved hemodynamic response function. FSL clustering features consider the spatial smoothing introduced to adequately adjust for multiple comparisons. Similar to established methods in the literature (Khoo et al., 2017; Wilson et al., 2024; Fahoum et al., 2012; Bénar et al., 2006; Bagshaw et al., 2004; An et al., 2013; Saad et al., 2001), the four z-score threshold BOLD response maps generated were amalgamated into a single cluster map by selecting the highest z-score for each voxel location from amongst the four z-score. Cluster descriptive statistics (e.g., centre of gravity, max z-score, extent) were generated for the final amalgamated cluster map using FSL cluster (Woolrich et al., 2009; Jenkinson et al., 2012).

### 2.5 Cluster definition and selection

For each analysis, the BOLD cluster with the highest positive and highest negative z-score were identified and deemed the maximum positive and maximum negative BOLD response, respectively. For each analysis, the cluster with the greatest z-score (regardless of the polarity, e.g., positive or negative) is designated the absolute maximum for that IED. For example, if the maximum positive and negative BOLD response z-score was 4.6 and 3.5 for a subject, respectively, then the positive cluster would be absolute maximum BOLD response. In contrast, if the maximum positive and negative BOLD response z-score was 3.5 and 4.6, respectively, then the negative cluster would be absolute maximum BOLD response. Note that in some patients, only a positive or only a negative BOLD response was observed. In those cases, the observed maximum BOLD response was deemed the absolute maximum BOLD response.

### 2.6 Electrode pairing

Some participants included in the study had multiple IED-generating regions (i.e., IED types) and more than one SOZ. Since both the IED-generating regions and the clinically determined SOZs are potential markers of the epileptogenic zone, we measured the distances between the IED-associated BOLD clusters and i) IED contacts and ii) SOZ contacts. IED types and SOZs were then paired based on proximity, pairing first based on shared electrodes and then on nearest electrode. Importantly, each IED type and SOZ could not be included in more than one pair. All unpaired SOZs were removed from further analyses.

### 2.7 Distance measurements

Euclidean distance was calculated between the geometric centre of the most active contact(s) for each IED type and the maximum voxel of the identified positive and negative BOLD clusters (*Equation (2)*). This calculation was repeated to measure the distance from the geometric centre of a clinically defined SOZ to the maximum voxel of the identified positive and negative BOLD clusters.

*Equation (2):* 3D distance equation

$${Distance}_{12}=\sqrt{{\left(x_{2}-x_{1}\right)}^{2}+{\left(y_{2}-y_{1}\right)}^{2}+{\left(z_{2}-z_{1}\right)}^{2}}$$

### 2.8 Statistical calculations

Statistical testing was performed using R within RStudio (R Core Team, 2022; RStudio, 2022). Linear mixed models were employed to examine the relationship between the distance measurements to the geometric centre of the IED contact(s) with a main effect of cluster polarity (maximum positive or maximum negative BOLD), a main effect of the absolute maximum (whether the absolute maximum is positive or negative) and an interaction, to determine if the effect of cluster polarity on distance depended on whether the absolute maximum was positive or negative. A random intercept was allowed by participant, to account for the presence of multiple IED types per participant. This model was repeated for distance to the geometric centre paired SOZ.

### 2.9 Default mode network

To study the relationship between the location of negative BOLD clusters and the DMN, we further restricted the analysis to only participants who did not have a prior surgery within the area of the DMN (n = 9), and participants and those in which the entire DMN was not captured in the MRI field of view (n = 10). A total of 38 participants were included in this portion of the analysis.

A region of interest (ROI) mask was created in each patient's functional space for the maximum positive and negative BOLD clusters. A neuroanatomical mask of the DMN (as described by Alves et al. (2019)) was registered to their functional space. The average fMRI time course from each ROI (DMN, maximum positive BOLD response, and maximum negative BOLD response) was calculated.

Pearson's correlation coefficients were calculated between the maximum negative BOLD responses and the DMN as well as between the maximum positive BOLD responses and the DMN to determine if these regions were functionally connected.

The spatial concordance between the maximum BOLD clusters and the DMN was also determined by first evaluating the number of voxels within the maximum BOLD cluster that overlapped with the voxels of the DMN – this was called the number of ‘overlap’ voxels. Subsequently, the number of overlap voxels was then divided by the total number of voxels of the maximum BOLD cluster to determine the percent of the maximum BOLD cluster that were within the DMN.

To statistically evaluate the relationship between positive and negative BOLD clusters and the DMN, we created a linear mixed model predicting functional connectivity with cluster polarity as a fixed effect, while including a random intercept for participants to account for the presence of multiple IED types per participant. This model was repeated for percent overlap.

## 3 Results

### 3.1 Participant characteristics

Participants were monitored by a physician during simultaneous iEEG-fMRI data acquisition and no ictal or adverse events occurred. Of the 70 participants, 5 were excluded due to inability to adequately remove gradient switching artifact from the iEEG, 5 were excluded from the analysis as they had less than 5 min of fMRI data after artifact and motion correction, and 3 participants failed to produce statistically significant BOLD clusters. Following these exclusions, 57 participants remained in the study, of which 26 (45.6%) were female, with an average age of 35 years (range 20-63 years), and disease duration of 18.0 years (range 2-53 years). In addition, 41 (71.9%) participants were diagnosed with temporal lobe epilepsy, and 23 (40.4%) had lesional MRI scans. For the DMN portion of the study, 10 participants were excluded because the DMN was not completely captured within the field-of-view on MRI, and an additional 9 had prior surgery, leaving 38 participants for that portion. See Table 1 for summary demographics.

**Table 1:** **Summary of clinical and demographic information.** Abbreviations: bFLE bilateral frontal lobe epilepsy, bTLE bilateral temporal lobe epilepsy, DMN default mode network, LFLE left frontal lobe epilepsy, LTLE left temporal lobe epilepsy, MFLE mesial frontal lobe epilepsy, n number, RFLE right frontal lobe epilepsy, RTLE right temporal lobe epilepsy, SD Standard Deviation, SOZ seizure onset zone.

	Overall	DMN Study
n	57	38
Sex (Female n (%))	26 (45.6)	18 (47.4)
Age at time of study, years (mean (SD))	34.98 (11.7)	34.84 (11.9)
Epilepsy onset age, years (mean (SD))	17.02 (11.1)	17.16 (11.4)
Disease Duration (mean (SD))	17.96 (12.92)	17.68 (12.26)
Handedness (n (%))		
Ambidextrous	5 (8.77)	3 (7.89)
Left	5 (8.77)	3 (7.89)
Right	47 (82.5)	32 (84.2)
Diagnosis (n (%))		
bTLE	13 (22.8)	9 (23.7)
LTLE	14 (24.6)	8 (21.1)
RTLE	14 (24.6)	10 (26.3)
bFLE	2 (3.5)	1 (2.6)
LFLE	5 (8.8)	3 (7.9)
RFLE	2 (3.5)	2 (5.3)
MFE	1 (1.8)	1 (2.6)
Other	6 (10.5)	4 (10.5)
Seizure frequency per month (mean (SD))	18.59 (35.3)	18.75 (28.7)
MRI lesion (n ‘Yes’ (%))	23 (40.4)	13 (34.2)
Previous surgery (n ‘Yes’ (%))	9 (15.8)	0 (0.0)
Number of IED types (n (%))		
1	21 (36.8)	12 (31.6)
2	26 (45.6)	21 (55.3)
3	6 (10.5)	2 (5.26)
4	3 (5.26)	2 (5.26)
5	1 (1.75)	1 (2.63)
Number of SOZ (n (%))		
1	36 (63.2)	23 (60.5)
2	18 (31.6)	13 (34.2)
3	3 (5.26)	2 (5.26)

### 3.2 Cluster selection

**Fig. 1:**
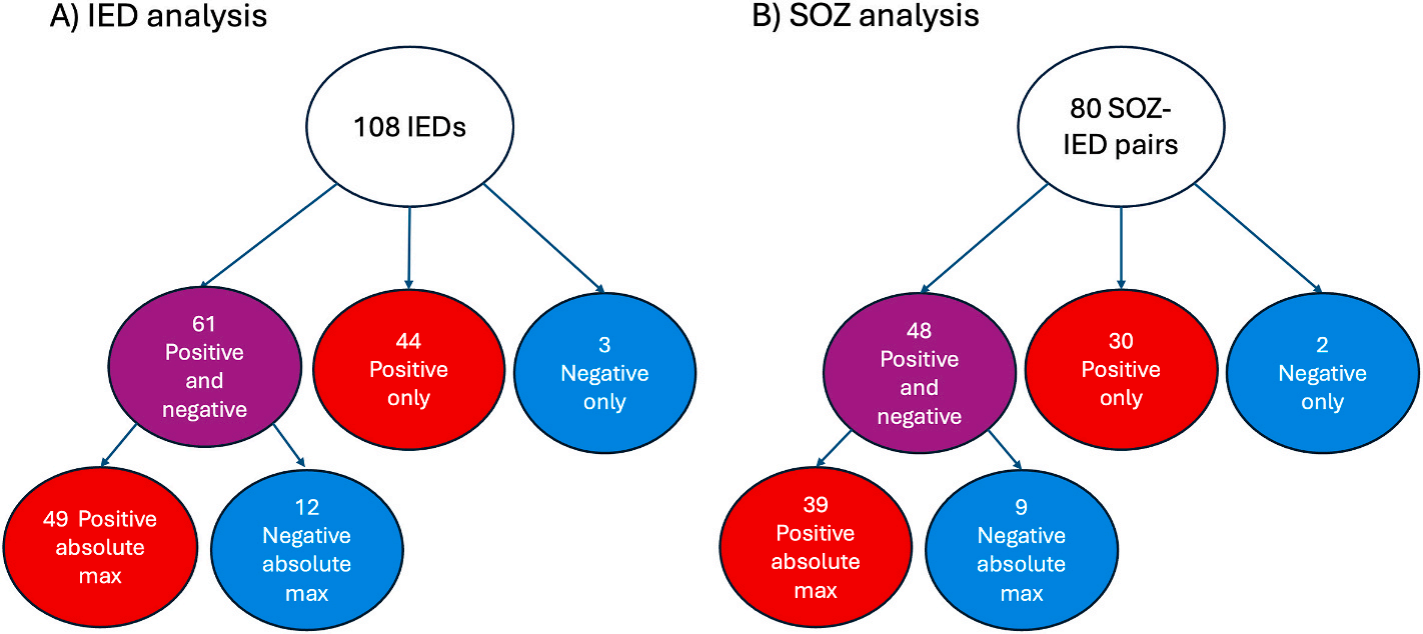
**Breakdown of cluster polarity and absolute maximum included in analyses.** A) summary of IEDs and B) summary of SOZ-IED pairs. Of note, in the instance of an IED or SOZ having both positive and negative clusters, where positive or negative is the absolute maximum, there are an equivalent number of negative or positive clusters, respectively, that are not the absolute maximum. As such, there are 105 maximal positive clusters (61 + 44), and 64 maximal negative clusters (61 + 3) in the IED analysis and 78 maximal positive clusters (48 + 30) and 50 maximal negative clusters (48 + 2) in the SOZ analysis. Abbreviations: IED interictal discharges, SOZ seizure onset zone.

**Fig. 2:**
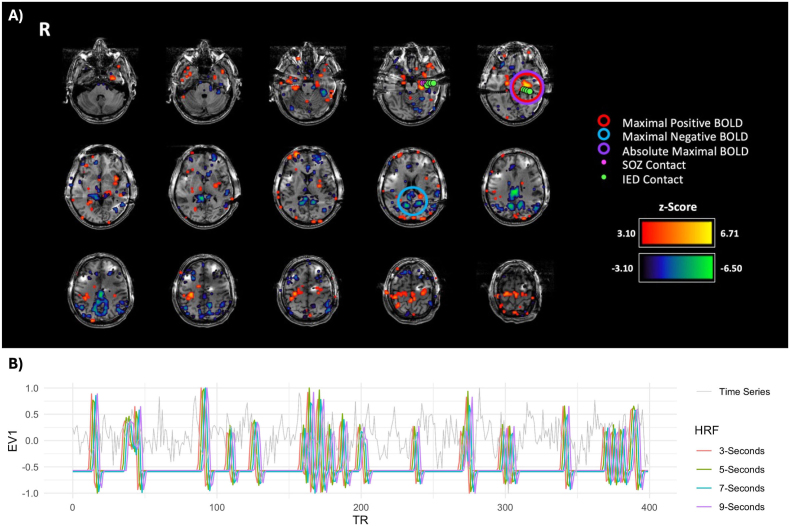
**iEEG-fMRI results for participant 38.** A) This exemplar demonstrates the typical finding of the maximal positive BOLD cluster being proximal to both the IED and SOZ EEG electrode contacts and the maximal negative BOLD cluster being distal to both. Additionally, for this subject, the maximal positive BOLD cluster was also the absolute maximal cluster which was the most common configuration in the cohort. Abbreviations: IED interictal discharges, SOZ seizure onset zone. B) fMRI models and time series results for participant 38. This exemplar demonstrates an excerpt of 400 TRs (10 min) for the four HRF models that were used to create fMRI BOLD maps for this participant, along with the normalized time series. Abbreviations: HRF hemodynamic response function, IED interictal discharges, SOZ seizure onset zone.

The average number of discharges identified per IED type was 468 (range = 5-2596) with participants having between 1 and 5 IED types and 1-3 SOZs. A total of 108 IED types were identified in 57 participants (Fig. 1). A total of 216 fMRI analyses were performed (i.e., positive and negative analyses for each IED), resulting in 169 BOLD maps with at least one significant cluster. Of these, 61 had both maximum positive and maximum negative clusters (i.e., 122 clusters), 44 had only positive clusters (i.e., 44 had no significant negative clusters), and 3 had only negative clusters (i.e., 3 had no significant positive clusters). Of the 61 IED types with both positive and negative maximum BOLD clusters, there were 49 instances where the positive cluster was also the absolute maximum. The absolute maximum cluster for IEDs that generated only positive or negative cluster maps was identified as the positive or negative maximum cluster, respectively. Overall, the absolute maximum was identified as the positive cluster for 93/108 (86.1%) IED types. A representative example of IED-associated BOLD activation maps is shown in Fig. 2A. Fig. 2B shows an excerpt from the same patient's time series plotted with the 4 HRFs used to make the composite maps.

A total of 80 SOZ-IED pairings were identified from 57 participants (Fig. 1). A total of 160 fMRI analyses were performed (i.e., positive and negative analyses for each SOZ-IED pair), resulting in 128 BOLD maps with at least one significant cluster. Of these, 48 had both maximum positive and maximum negative clusters (i.e., 96 clusters), 30 had only positive clusters (i.e., 30 had no significant negative clusters), and 2 had only negative clusters (i.e., 2 had no significant positive clusters), for a total of 128 clusters. Of the SOZ-IED pairings that generated both positive and negative maximum BOLD clusters, there were 39 instances where the positive cluster was also the absolute maximum. SOZ-IED pairings that generated only positive or negative clusters were considered to have a positive or negative absolute maximum, respectively. Overall, the absolute maximum BOLD cluster was positive for 69/80 SOZ-IED pairs.

### 3.3 Distance analyses

**Fig. 3:**
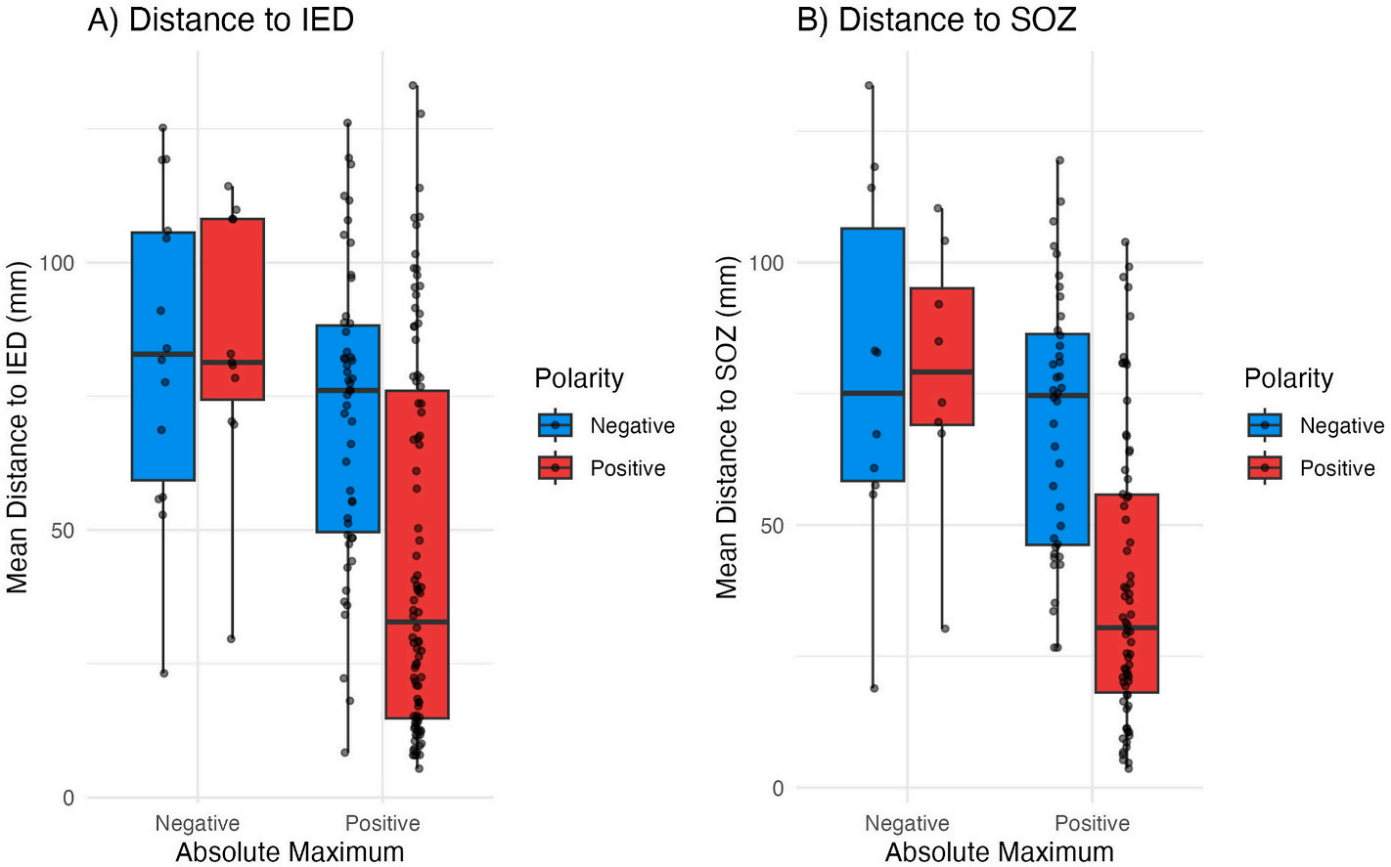
**Boxplots of distance from positive and negative BOLD clusters to epileptic tissues**. A) Distance from positive and negative BOLD clusters to the IED generating contact(s) when the absolute maximum is positive and negative. B) Distance from positive and negative BOLD clusters to the SOZ when the absolute maximum is positive and negative. Abbreviations: IED interictal epileptiform discharges, SOZ seizure onset zone.

We created a linear mixed model of the effect of cluster polarity, absolute maximum, and the interaction between cluster polarity and the absolute maximum, on distance to the IED generating contact(s). The interaction was significant (Estimate = −30.0, 95% CI [−55.5, −4.36], Fig. 3A), indicating that positive BOLD clusters are closer to the IED generating contact(s) than negative BOLD clusters, specifically when the absolute maximum is positive (positive BOLD clusters: Median = 31.75 mm, IQR = [14.75, 73.65], negative BOLD cluster: Median = 73.27 mm IQR = [49.07,87.12]). There was no discernible difference between positive and negative BOLD clusters when the absolute maximum was negative (positive BOLD clusters: Median = 82.13 mm, IQR = [76.38, 108.1], negative BOLD cluster: Median = 81.80 mm IQR = [62.42, 105.2]). The main effects of cluster polarity and absolute maximum were not significant.

We created a linear mixed model of the effect of cluster polarity, absolute maximum, and the interaction between cluster polarity and the absolute maximum on distance to the SOZ. The interaction was significant, (Estimate = −32.7, 95% CI [−57.5 -16.5], Fig. 3B) indicating that positive BOLD clusters are closer to the IED generating contact(s) than negative BOLD clusters, specifically when the absolute maximum is positive (positive BOLD clusters: Median = 29.91 mm, IQR = [17.71, 55.50], negative BOLD cluster: Median = 62.29 mm IQR = [46.17, 82.24]). Again, there was no discernible difference between positive and negative BOLD clusters when the absolute maximum was negative (positive BOLD clusters: Median = 84.99 mm, IQR = [69.60, 95.00], negative BOLD cluster: Median = 79.62 mm IQR = [52.33, 98.71]). The main effects of cluster polarity and absolute maximum were not significant.

### 3.4 Cluster DMN analysis

We created a linear mixed model predicting functional connectivity with the DMN including a main effect of cluster polarity and a random intercept for participant and did not find a difference in functional connectivity for positive and negative BOLD clusters to the DMN. Evaluation of the marginal means for this model reveals that neither the functional connectivity for negative BOLD clusters (marginal mean = 0.180, 95% CI [−0.009, 0.370]) or positive BOLD clusters (marginal mean = 0.122, 95% CI [−0.030, 0.276] are significantly different from zero.

**Fig. 4:**
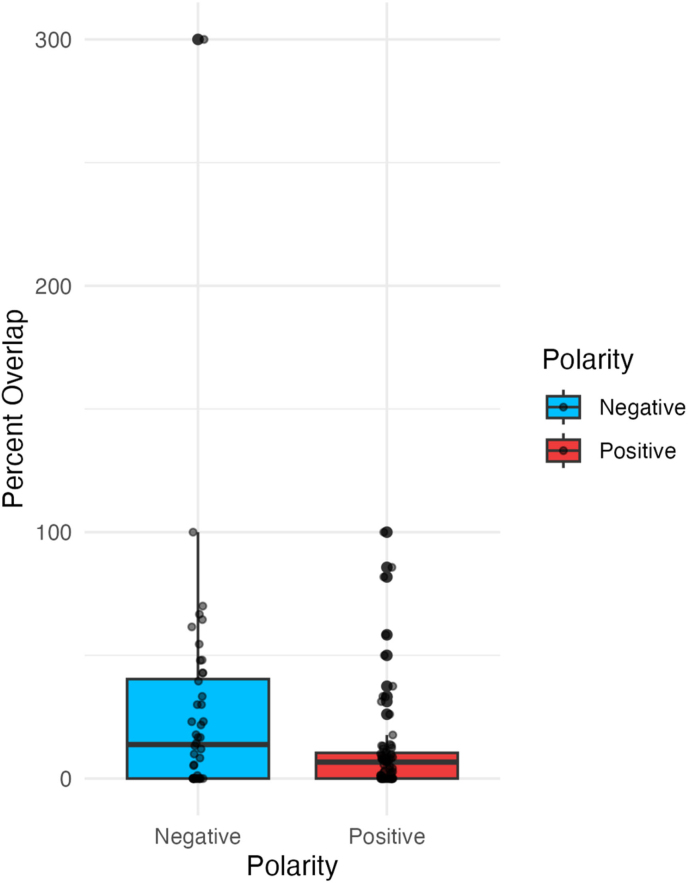
**DMN analysis.** Boxplots of spatial overlap with the DMN, expressed as % of BOLD cluster voxels that overlap with the DMN, for positive and negative clusters.

We also created a model predicting clusters’ percent overlap with the DMN, including a main effect of cluster polarity and a random intercept for participant and found that negative clusters (mean % = 27.7%) have significantly greater overlap with the DMN than positive clusters (mean % = 11.4%) (Estimate = −16.3, 95% CI [−29.0, −3.72], Fig. 4). However, this model was found to have singular fit which can be considered untrustworthy. To address this concern, we selected positive and negative clusters from only the most clinically relevant IED per participant, as determined by the epileptologists who reviewed the EEG. This method resulted in a single positive and negative cluster for 22 participants, for which proportion overlap with the DMN was assessed using paired t-tests. We found that the proportion of a cluster that overlaps with the DMN was significantly greater for negative BOLD clusters (M = 0.225, SD = 0.215) than for positive BOLD clusters (M = 0.100, SD = 0.161) (T (21) = −2.14, p = 0.04, Fig. 4). Note that a paired *t*-test such as this is more accurately represented by examining difference scores between positive and negative BOLD clusters and thus can be significant even if overlap exists among confidence intervals.

### 3.5 Removing negative BOLD responses that overlap with the DMN

**Supplementary Fig. 1:**
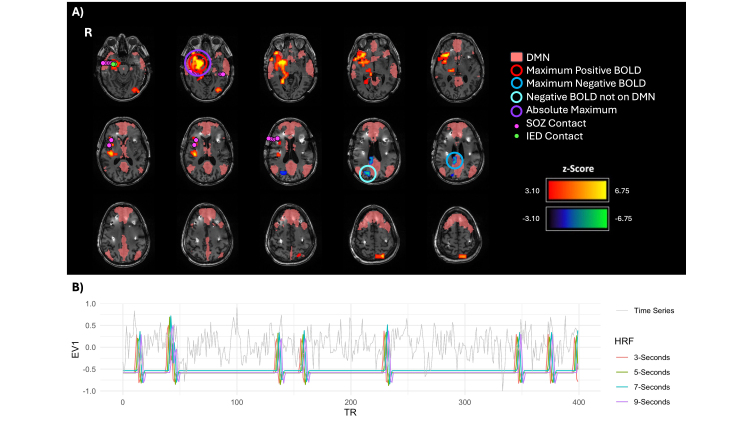
**iEEG-fMRI results for participant 43.** A) This exemplar demonstrates the maximal positive and negative bold clusters relative to the IED and SOZ contacts, and the DMN mask registered to this participant. In this instance, the maximal negative BOLD cluster overlapped with the DMN. The next highest z-score not overlapping with the DMN is also shown. Abbreviations: IED interictal discharges, SOZ seizure onset zone, DMN default mode network. B) This exemplar demonstrates an excerpt of 400 TRs (10 min) for the four HRF models that were used to create fMRI BOLD maps for this participant, along with the normalized time series. Abbreviations: HRF hemodynamic response function, IED interictal discharges, SOZ seizure onset zone, DMN default mode network.

We created a second dataset that excludes negative BOLD clusters overlapping with the DMN to compare our findings to scalp EEG-fMRI studies that do the same (Khoo et al., 2017; Koupparis et al., 2021). A representative example of a participant with maximum negative BOLD clusters in the DMN and the highest z-score alternative can be seen in Supplementary Fig. S1. Using the spatial concordance calculated for the DMN analysis, we identified 24 out of 64 IED-types with negative BOLD clusters that had more than 10% of voxels overlapping with the DMN. We were able to identify a replacement negative BOLD cluster that did not overlap with the DMN for 12 of the 24 identified IED-types, hence excluding 12 IED-types, leaving 52 IED-types. In addition, of the 52 remaining IED-types, the negative BOLD response from 20 more IED-types were from 19 participants in which the entire DMN was not captured in the MRI field of view or who had prior surgery (i.e., were excluded from the DMN study) as we could not verify whether the cluster overlapped with the DMN. This left negative BOLD responses to 32 IED-types available for this analysis. All positive clusters remained in the study (n = 105). The polarity of the absolute maximum was reevaluated for IED-types that had negative BOLD clusters changed or excluded.

Following these additional exclusions, 56 participants remained in the study. A total of 105 IED-types were considered. Of these 32 IED-types had both positive and negative BOLD clusters and 73 had only positive BOLD clusters. The absolute maximum was negative for 4 IED-types, as compared to the 101 times the absolute maximum was positive. A total of 78 SOZ-IED pairs were included. Of these, 28 SOZ-IED pairs had both positive and negative BOLD clusters, and 50 had only positive BOLD clusters. The absolute maximum was negative for only 4 SOZ-IED pairs as compared to 74 SOZ-IEDs for which the absolute maximum was positive.

We recreated the linear mixed models of the effect of cluster polarity, absolute maximum, and the interaction between cluster polarity and the absolute maximum, on distance to the IED generating contact(s) and the SOZ. For both analyses, the models were found to have singular fit indicating that our data did not support the models’ complexity, and the interaction between polarity and absolute maximum was non-significant. Simplifying the models to include only the effect of cluster polarity resulted in properly fitted models revealing that positive BOLD responses were closer to the IED generating contact(s) (positive BOLD clusters: Median = 38.22 mm, IQR = [15.31, 78.98], negative BOLD clusters: Median = 76.75 mm IQR = [54.49, 88.66]) (Estimate = −25.89, 95% CI = [−39.24, −12.48]) and the SOZ (positive BOLD clusters: Median = 32.69 mm, IQR = [20.10, 66.23], negative BOLD clusters: Median = 74.31 mm IQR = [52.52, 86.37]) (Estimate = −24.502, 95%CI = [−38.72, −14.90])

## 4 Discussion

In this study, we examined positive and negative BOLD responses to IEDs using iEEG-fMRI in patients with drug-resistant focal epilepsy. Our findings demonstrate that maximum positive BOLD clusters are significantly closer to both the IED-generating contacts and the SOZ compared to the maximum negative BOLD clusters and this effect persisted when negative BOLD clusters overlapping with the DMN were removed. We further found that positive clusters were significantly closer to IED generating contacts and the SOZ only when the positive cluster is the absolute maximum. In contrast, maximal negative BOLD clusters were not closer to the IED-generating contacts or the SOZ, even when these clusters were the absolute maximum. Additionally, we found that negative BOLD clusters show greater spatial overlap with the DMN than positive clusters, though functional connectivity with the DMN did not differ significantly between positive and negative cluster types.

The consistent finding that maximum positive clusters are closer to both IED-generating contacts and the SOZ than maximum negative clusters support the current understanding that positive BOLD responses reflect increased neural activity in epileptogenic tissue. This finding contrasts previous scalp EEG-fMRI studies which suggest the absolute maximum cluster (regardless of polarity) can be used for SOZ localization (Koupparis et al., 2021; Khoo et al., 2018). In our study, the absolute maximum cluster was positive in approximately 84% of cases and when considered all together, we found that positive clusters were closer than negative clusters. However, when we considered the polarity of the cluster with the polarity of the absolute maximum, we found that the interaction between absolute maximum and individual cluster polarity was significant, suggesting that the effect of polarity on distance to the IED generator or SOZ differs based on whether the cluster is the absolute maximum. We would expect this finding if negative clusters had some localizing value when the negative cluster is the absolute maximum, but in our data, it does not appear that negative BOLD is closer to the IED generator or SOZ, but that both positive and negative clusters perform poorly when the absolute maximum is negative. The inferior localization performance of negative BOLD clusters aligns with previous literature suggesting that negative BOLD responses often localize to regions outside epileptogenic tissue (Kobayashi et al., 2006b). Our findings suggest that including the negative clusters by using the absolute maximum BOLD cluster is a less reliable indicator of the IED generator and the SOZ.

Our finding that negative BOLD clusters show greater spatial overlap with the DMN than positive clusters provide important insights into the neurobiological basis of negative BOLD responses in epilepsy. The DMN, which includes regions such as the medial prefrontal cortex, posterior cingulate cortex, and angular gyrus, is known to be active during rest and deactivated during task-focused states (Menon, 2023). The preferential overlap of negative BOLD clusters with DMN regions suggests that these responses may reflect deactivation of the task-negative network during the timing of epileptiform discharges rather than direct epileptogenic activity.

This interpretation is consistent with the “network inhibition” hypothesis (Norden and Blumenfeld, 2002), which posits that epileptiform activity in focal regions can lead to widespread deactivation of distant brain networks, particularly the DMN. Indeed, negative BOLD in the DMN has been found to be more related to task performance than to functional connectivity (Parker and Razlighi, 2019). The lack of significant difference in functional connectivity between positive and negative clusters with the DMN, despite the spatial overlap differences, may indicate that the relationship between negative BOLD responses and the DMN is more complex than simple functional coupling.

### 4.1 Limitations

Several methodological factors may have influenced our findings. Firstly, our sample is limited in the clusters produced. Only 61/108 IEDs and 48/80 IED-SOZ pairs had both positive and negative BOLD clusters for comparison and only 15/108 IEDs and 11/80 IED-SOZ pairs had the greatest Z-score for negative BOLD. It is possible that a larger sample would be able to draw out more subtle differences in the effect of cluster polarity when the absolute maximum Z-score is negative.

Secondly, the use of intracranial electrodes, while providing superior EEG signal quality and IED detection compared to scalp EEG, creates significant MRI artifacts that may obscure BOLD signals in proximity to the electrodes (Boucousis et al., 2012). This artifact could potentially bias our distance measurements, though it would likely affect both positive and negative clusters similarly. Second, our analysis utilized conventional hemodynamic response functions (HRFs) with time-to-peak offsets of 3, 5, 7, and 9 s. Recent research has identified negative BOLD-specific HRFs (Suarez et al., 2021) that may differ from canonical positive responses, potentially in timing, amplitude, or spatial extent. The use of conventional HRFs may have limited our ability to detect and characterize negative BOLD responses, possibly leading to underestimation of their clinical utility. Finally, the hierarchical nature of our data (multiple IED types and SOZs nested within participants) required careful statistical modeling. While our linear mixed models appropriately accounted for within-participant correlations, the complexity of the data structure may have limited our power to detect subtle effects.

### 4.2 Conclusion

This study provides important evidence that positive BOLD responses to IEDs are superior to negative responses for localizing epileptogenic tissue in patients with drug-resistant focal epilepsy. While negative BOLD responses show interesting relationships with the default mode network, they appear to be less clinically relevant for presurgical planning. Our findings suggest that clinical interpretation of EEG-fMRI should continue to prioritize positive BOLD responses, while negative responses should be interpreted with caution and not considered equivalent indicators of epileptogenic activity. The study also highlights the complexity of negative BOLD responses in epilepsy and the need for continued research to fully understand their neurobiological basis and potential clinical applications. As methodological advances continue to improve our ability to detect and characterize negative BOLD responses, their role in epilepsy evaluation may become clearer, but current evidence supports the continued emphasis on positive BOLD responses for clinical decision-making.

## Funding

Canadian Institute of 10.13039/100005622Health Research (MOP-230809).

## CRediT authorship contribution statement

**Jessie Hart Szostakiwskyj:** Data curation, Formal analysis, Methodology, Writing – original draft, Writing – review & editing. **Victoria Mosher:** Conceptualization, Data curation, Formal analysis, Methodology, Writing – original draft, Writing – review & editing. **Perry Dykens:** Data curation, Investigation, Methodology. **William Wilson:** Conceptualization, Investigation, Methodology, Writing – review & editing. **Yi Fan Qi:** Data curation, Investigation. **Dan J. Pittman:** Conceptualization, Data curation, Formal analysis, Investigation, Methodology, Software. **Laura Gill:** Investigation, Writing – review & editing. **Joseph Peedicail:** Data curation, Investigation. **Brad G. Goodyear:** Conceptualization, Data curation, Formal analysis, Supervision, Writing – review & editing. **Paolo Federico:** Conceptualization, Funding acquisition, Project administration, Supervision, Validation, Writing – review & editing. **Yahya Aghakhani:** Data curation. **Walter Hader:** Data curation. **Nathalie Jetté:** Data curation. **Colin Josephson:** Data curation. **Karl Martin Klein:** Data curation. **William Murphy:** Data curation. **Neelan Pillay:** Data curation. **Andrea Salmon:** Data curation. **Shaily Singh:** Data curation. **Yves Starreveld:** Data curation. **Samuel Wiebe:** Data curation.

## Declaration of competing interest

XX The authors declare that they have no known competing financial interests or personal relationships that could have appeared to influence the work reported in this paper.

## Data Availability

Data will be made available on request.
